# The glyconanoparticle as carrier for drug delivery

**DOI:** 10.1080/10717544.2018.1519001

**Published:** 2018-12-26

**Authors:** Xueqin Zhang, Gangliang Huang, Hualiang Huang

**Affiliations:** a Active Carbohydrate Research Institute, Chongqing Normal University, Chongqing, China;; b School of Chemistry and Environmental Engineering, Wuhan Institute of Technology, Wuhan, China

**Keywords:** Glyconanoparticle, carrier, drug delivery, application

## Abstract

The glyconanoparticle (GlycoNP) has multiple effects and has important applications in drug delivery and bioimaging. It not only has the advantages of nano drug delivery system but also utilizes the characteristics of multivalent interaction of sugar, which greatly improves the targeting of drug delivery. Herein, the application of GlycoNP in drug delivery was analyzed and discussed, the solution to its problem was proposed, and its prospects were forecasted.

## 1. Introduction

Carbohydrate is the most abundant biological molecule in nature, and it is also a basic element of a series of life processes in living organisms (Fasman, 1975). In addition to being able to make up the structure of living organisms and provide energy for them, carbohydrates can also widely mediated the recognition process in living systems through their interactions with proteins, nucleic acids, lipids, and other molecules, such as cell communication and migration (Grillon et al., 2012), tumor production and development (Hakomori, 2001), immune response (Nieman, 1998), fertilization (Töpfer-Petersen, 1999), apoptosis and infection (Mirkin, 1973). However, there are many difficulties in the study of these processes, mainly in the following two aspects. One is that the carbohydrate chains involved in these processes are complex in structure and have a low content in cells. The second is that the biological effects associated with carbohydrate are often very low and difficult to monitor. However, with the great development of carbohydrate synthesis technology, carbohydrate chain analysis methods and nanotechnology, research on the biological effects associated with carbohydrate has become a hot topic in recent years.

Nanomaterials as the carriers of carbohydrates have been gradually developed since the first synthesis of carbohydrate-functionalized gold nanoparticles in 2001 (de la Fuente et al., 2001), and related reports have gradually increased. They have shown great potential for applications in biomedical imaging, diagnosis, and treatment (García et al., 2010). Compared with the use of other molecules as carriers, nanoparticles can regulate the density of ligands on the surface by adjusting their size and shape. In addition, nanoparticles have unique optical, electrical, magnetic, mechanical, and chemical activities. These properties make glyconanoparticles not only useful for studying sugar-related biological effects but also for cell imaging and tumor cell-targeted drug delivery (Jafari et al., 2014). Herein, the nanoparticles of important monosaccharides and oligosaccharides during life were summarized, and then the applications of glyconanoparticles were discussed and analyzed.

## 2. Applications of glyconanoparticles

As mentioned in the introduction, carbohydrates are generally weak in their action, but it can be compensated by multiple ligands. The discovery of the multivalent effect of carbohydrate–lectin interaction greatly promoted the development of glyconanomaterials. One method is to assemble many carbohydrate molecules onto the surface of nanoparticles. First of all, this way of presenting multiple ligands to interact with proteins greatly enhances the interaction between them. Second, glyconanoparticles are in the same size range as many biomolecules. They can imitate the sugar coating on the cell surface and serve as a good model for biological cells. Finally, due to quantum size effect, glyconanoparticles have unusual physical properties that can be used to specifically probe these interactions. These three properties of glyconanoparticle make it a great advantage in the study of sugar-sugar interactions and sugar-protein interactions. The glyconanoparticles have been widely used in biomarkers and biomedicines (Marradi et al., 2010).

### 2.1. Glyco-quantum dots

Since glyconanoparticles can constitute a good model for simulations to intervene in carbohydrate-based life processes, they have been tried in biopharmaceuticals. A novel type of quantum dots (QDs) that wrapped carbohydrate molecules was reported, they could specifically bind to cell surface receptors in certain tissues and organs. Certain liver cells had asialoglycoprotein receptor on their surfaces. It could specifically bind to QDs with galactose residues (Yang et al., 2010). Further studies showed that HepG2 cells were preferentially filled with galactose-QDs via receptor-mediated endocytosis. In mouse experiments, QDs of d-mannose and d-galactose were found to selectively accumulate in mouse hepatocytes. The carbohydrate-encapsulated QDs were concentrated in the liver of mice. The degree was three times that of ordinary QDs, namely the former was more selective. The results showed that the carbohydrate-coated QDs had good potential for *in vivo* targeting.

**Figure 1.**
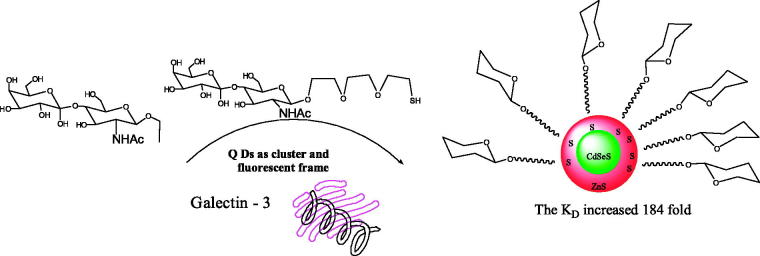
The interactions of quantum dots and galectin-3.

Yang et al. (2012) have prepared *N*-acetyl lactosamine, bivalent *N*-acetyl lactosamine, and *N*-acetyl lactosamine coated QDs as a fluorescent probe to study the interactions of *N*-acetyl lactosamine and galectin-3 (Figure 1).

Moreover, Yang et al. (2010) prepared a series of lactose-CdSeS/ZnS QD conjugates with different lactose densities and a PEGylated (*n* = 3) lactose-QDs conjugate with more flexible sugar ligands.

A versatile method has been developed for highly sensitive and selective *in situ* evaluation of cell surface sialic acid (SA) groups by combining the multiplex sandwich binding of the 3-aminophenylboronic acid functionalized QD (APBA-QD) probes to SA groups on living cells, glyconanoparticles, and the sensitive fluorescence detection of metal-responsive dye (Liu et al., 2013).

Kikkeri and Bavireddi (2012) have shown a host–guest approach to prepare water-soluble glyco-QDs by using *O*-α-manno- and *O*-β-galactopyranoside capped β-CD as surface-coating agents.

Jiang et al. (2009) have successfully synthesized biotin and carbohydrate functionalized reagents for the surface functionalization of QDs.

A versatile method has been developed for highly sensitive and selective *in situ* evaluation of cell surface SA groups by combining the multiplex sandwich binding of the APBA-QD probes to SA groups on living cells, glyconanoparticles, and the sensitive fluorescence detection of metal-responsive dye (Han et al., 2011).

### 2.2. Gold/silver glyconanoparticles

Nanotechnology offers new tools to improve HIV drug treatment and prevention. The gold nanoparticles are an interesting chemical tool to design and prepare smart and efficient drug-delivery systems. The pH-mediated release of drugs from the glyconanoparticles has been determined. It proved that the gold glyconanoparticles were a new multifunctional drug-delivery system in the therapy against HIV (Chiodo et al., 2014).

Dendritic cell-based (DC-based) vaccines are promising immunotherapies for cancer. However, the lack of efficient targeted delivery and the sources and types of DCs has limited the efficacy of DCs and their clinical potential. Calderon-Gonzalez et al. (2017) proposed an alternative nanotechnology-based vaccine platform with antibacterial prophylactic abilities, which used gold glyconanoparticles coupled to listeriolysin O 91–99 peptide (GNP-LLO_91–99_). GNP-LLO_91–99_ exhibited dual antitumour activities. It was proposed this adjuvant nanotherapy for preventing the progression of the first stages of melanoma.

Gold glyconanoparticles (GNPs) are full of promise in areas like biomedicine, biotechnology and materials science because of their amazing physical, chemical and biological properties. *In vivo* pulmonary delivered siRNA GNPs were capable of targeting c-Myc gene expression via *in vivo* RNAi in tumor tissue, which led to an ∼80% reduction in tumor size without associated inflammation (Conde et al., 2015).

The human galectin-3 (Gal-3) is well-known to be overexpressed in several human tumors and can act as a biorecognizable target. Aykaç et al. (2014) prepared the β-cyclodextrin-bearing gold glyconanoparticles for estimating their loading capability toward the anticancer drug methotrexate (MTX). It showed that these glyconanoparticles should be potential site-specific delivery systems for anticancer drugs.

Qian et al. (2017) reported a highly efficient transport nanostructure based on core-shell glyconanoparticles (GNPs), with gold as the core and dextran as the shell interspersed with metformin molecules. The dextran shell facilitated the entry of GNPs into living cells, which allowed the subsequent release of metformin. Compared with bare metformin or bare GNPs, magnetic glyconanoparticles (MGNPs) showed a stronger capacity for cell growth inhibition with good biocompatibility. This work provided a new therapeutic tool for the treatment of cancer.

**Figure 2.**
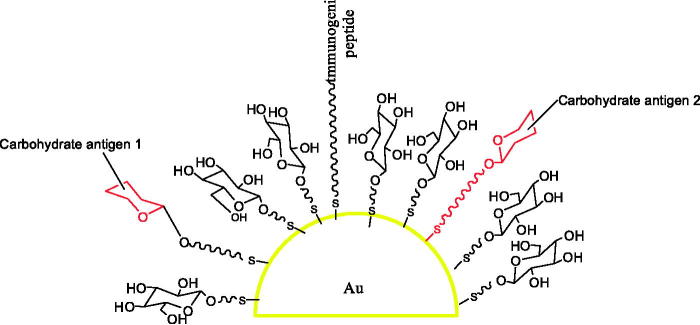
The gold glyconanoparticles for screening carbohydrate-based anticancer vaccines.

Ojeda et al. (2007) reported that 10 different multifunctional gold glyconanoparticles incorporating sialylTn and Lewis^y^ antigens, T-cell helper peptides, and glucose in well-defined average proportions and with different density were synthesized in a one-step procedure (Figure 2).

The researchers (Chiodo & Marradi, 2015) have found that gold nanoparticles have great potential as carriers for the development of a great diversity of fully synthetic carbohydrate-based vaccines.

In order to provide a means of detecting the bacterially secreted toxin, Schofield et al. (2007) developed a simple and rapid colorimetric bioassay for the cholera toxin.

**Figure 3.**
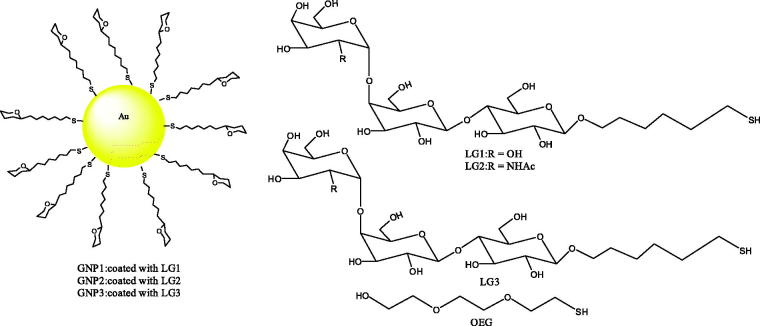
The structure of gold glyconanoparticle.

Kulkarni et al. (2010) developed gold glyconanoparticles that present a multivalent display similar to the cell surface glycolipids to compete for the selective inhibition of Shiga toxins (Stx) 1 and 2 (Figure 3).

Wang et al. (2013) reported a new type of glyconanoparticle microarray to study glycan-lectin interactions.

Arnáiz et al. (2012) prepared the fluorescent-labeled glyconanoparticles (FITC-*manno*GNPs) and studied their uptake by DC-SIGN expressing Burkitt lymphoma cells (Raji DC-SIGN cell line) and monocyte-derived immature dendritic cells.

Nagahori et al. (2009) communicated such an approach to a novel “omics”, namely, glycosphingolipidomics based on the “glycoblotting” method.

The Thomsen–Friedenreich antigen-containing glycopeptide thiols based on a mucin peptide repeating unit were prepared. These novel multivalent tools should prove extremely useful in exploring the binding properties and immune response to this important carbohydrate antigen (Sundgren & Barchi, 2008).

**Figure 4.**
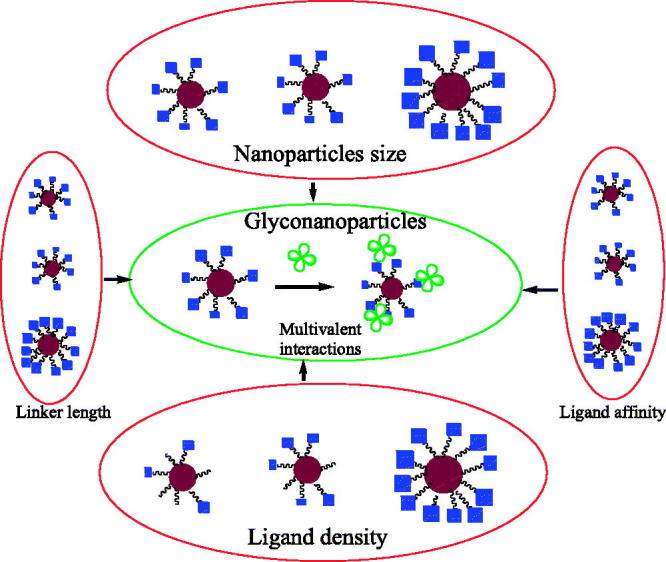
The factors affecting the binding affinity of glyconanoparticles.

Wang et al. (2010) reported a new method to measure the binding affinity of glyconanoparticle–protein interactions based on a fluorescent competition binding assay (Figure 4).

Carvalho et al. (2008) studied the gold nanoparticles coated with a pyruvated trisaccharide epitope of the extracellular proteoglycan of *Microciona prolifera* as potential tools to explore carbohydrate-mediated cell recognition.

### 2.3. Magnetic glyconanoparticles

Activation of the endothelium is a pivotal first step for leukocyte migration into the diseased brain. So, imaging this activation process is highly desirable. It indicated that the targeted carbohydrate-functionalized magnetic nanoparticles accumulated in the brain vasculature following acute administration into a clinically relevant animal model of stroke (Farr et al., 2014).

Dual-modal fluorescent magnetic glyconanoparticles are powerful in probing lectins displayed on pathogenic and mammalian cell surfaces. It indicated that glyconanoparticles were useful tools to enrich lectin expressing cells because of their magnetic properties. The dual-modal glyconanoparticles were biocompatible and that they could be employed in lectin-associated biological studies and biomedical applications (Park et al., 2015).

Magnetic glyconanoparticles were synthesized by the co-precipitation method, and they were formed in a simple and direct process (Kekkonen et al., 2009).

**Figure 5.**
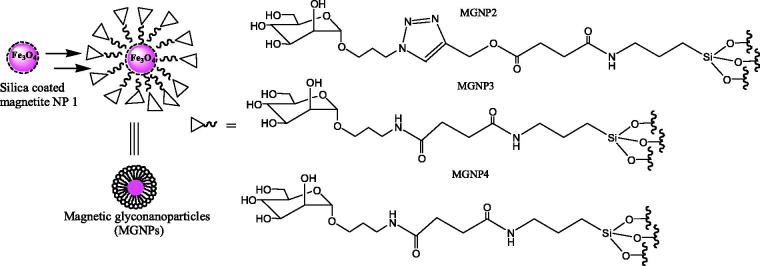
The magnetic glyconanoparticles.

El-Boubbou et al. (2007) demonstrated the potential of sugar-coated magnetic nanoparticles for fast bacterial detection and removal (Figure 5).

de la Fuente et al. (2006) reported a simple method for the preparation of gold–iron nanoparticles, which were protected and functionalized with biologically different carbohydrates (maltose, lactose, and glucose).

Sundhoro et al. (2017) reported a versatile approach for the immobilization of unmodified monosaccharides onto iron oxide nanoparticles, which were functionalized with phosphate-containing perfluorophenylazides coupling agents.

Gallo et al. (2011) offered an *in vitro* simple method to evaluate the efficiency of the magnetic probes to label specifically cell populations in the whole blood by magnetic resonance imaging and fluorescence techniques. In addition, García et al. (2011) also established a versatile platform of magnetic glyconanoparticles for selective immunolabeling and imaging of cells. Kasteren et al. (2009) designed and constructed a carbohydrate-functionalized nanoparticles that allowed pre-symptomatic *in vivo* imaging of brain disease.

Zhou et al. (2015) developed a stimuli-responsive drug delivery system based on fluorescent, lectin-gated, and mesoporous glyconanoparticles. The gating process was efficient, which exhibited good sealing properties in the absence of the glutathione redox trigger and avoided premature release in normal cells. The lectin gate was rapidly opened and the anticancer drug released in the presence of higher levels of glutathione in cancer cells.

Glyconanoparticles that exhibit multivalent binding to lectins are desirable for molecular recognition and therapeutic applications. Besford et al. (2017) explored the use of glycogen nanoparticles as a biosourced glycoscaffold for engineering multivalent glyconanoparticles. The galacto-glycogen nanoparticles induced the aggregation between prostate cancer cells. It highlighted a strategy for engineering a biosourced polysaccharide with surface moieties that exhibited strong multivalent interactions with lectins, and targeted interaction with prostate cancer cells.

Wu et al. (2017) explored the architectural effect of the glyconanoparticle corona on glyconanoparticle macrophage endocytosis and lectin-binding ability. Nanoparticles with a mixed shell showed a higher efficiency in cellular uptake and lectin-binding than those with a single sugar component. Moreover, homogeneously mixed coronas (MG) presented a significantly higher efficiency than blend-mixed coronas (M/G).

Gallegos-Tabanico et al. (2017) modified bovine serum albumin (BSA) with lactose, obtaining a neoglycan (BSA-Lac). Then they synthesized glyconanoparticles (NP-BSA-Lac). The results indicated that the lactosylated nanovectors could be targeted at the *E. coli* K88 adhesin and potentially could be used as a transporter for an antibacterial drug.

Won et al. (2018) introduced multivalent glyconanostructures, which enabled the lactose moieties to be presented only when an external stimulus was present, mimicking how nature used enzymes to dynamically regulate glycan expression.

## 3. Conclusion and future prospects

The targeted drug delivery has been investigated as one of the main methods in medicine to ensure successful treatments of diseases. Pharmaceutical sciences are using micro or nano carriers to obtain a controlled delivery of drugs, able to selectively interact with pathogens, cells or tissues.

Herein, the construction, preparation, and applications of several common water-soluble and stable glyconanoparticles were summarized and discussed. It can be clearly seen that these new multivalent systems have opened up new avenues for the study of carbohydrate-involved biological interactions. These glyconanoparticles are easy to prepare and have unique physical, chemical, and biological properties, which make them have a wide range of applications in drug delivery, biomedical imaging, diagnosis, and treatment. It is believed that with the in-depth study of carbohydrate nanobiology, as well as the crossing of chemistry, physics, and pharmacy, the research of carbohydrate QDs, gold/silver glyconanoparticles, and magnetic glyconanoparticles will make great progress, and find a wider application field.

Major effort should be focused toward the design and synthesis of more complex and biologically relevant carbohydrate mimics in order to have a better understanding of the carbohydrate–carbohydrate and carbohydrate–protein interactions. The full therapeutic potential of these carbohydrate-based nanoparticles systems can be achieved when the functions of carbohydrates in biological systems are clarified.

## References

[CIT0001] Arnáiz B, Martínez-Ávila O, Falcon-Perez JM, et al. (2012). Cellular uptake of gold nanoparticles bearing HIV gp120 oligomannosides. Bioconjug Chem 23:814–25. 22433013 10.1021/bc200663r

[CIT0002] Aykaç A, Martos-Maldonado MC, Casas-Solvas JM, et al. (2014). β-Cyclodextrin-bearing gold glyconanoparticles for the development of site specific drug delivery systems. Langmuir 30:234–42. 24313322 10.1021/la403454p

[CIT0003] Besford QA, Wojnilowicz M, Suma T, et al. (2017). Lactosylated glycogen nanoparticles for targeting prostate cancer cells. ACS Appl Mater Interfaces 9:16869–79. 28362077 10.1021/acsami.7b02676

[CIT0004] Calderon-Gonzalez R, Terán-Navarro H, García I, et al. (2017). Gold glyconanoparticles coupled to listeriolysin O 91-99 peptide serve as adjuvant therapy against melanoma. Nanoscale 9:10721–32. 28714508 10.1039/c7nr02494k

[CIT0005] Carvalho d. S A, Vliegenthart JF, Kamerling JP (2008). Gold nanoparticles coated with a pyruvated trisaccharide epitope of the extracellular proteoglycan of *Microciona prolifera* as potential tools to explore carbohydrate-mediated cell recognition. Org Biomol Chem 6:2095–102. 18528571 10.1039/b802235f

[CIT0006] Chiodo F, Marradi M, Calvo J, et al. (2014). Glycosystems in nanotechnology: gold glyconanoparticles as carrier for anti-HIV prodrugs. Beilstein J Org Chem 10:1339–46. 24991287 10.3762/bjoc.10.136 PMC4077455

[CIT0007] Chiodo F, Marradi M (2015). Gold nanoparticles as carriers for synthetic glycoconjugate vaccines In Carbohydrate-based vaccines, Lepenies B (Ed.). New York: Springer, 159. 10.1007/978-1-4939-2874-3_10 26169740

[CIT0008] Conde J, Tian F, Hernandez Y, et al. (2015). RNAi-based glyconanoparticles trigger apoptotic pathways for *in vitro* and *in vivo* enhanced cancer-cell killing. Nanoscale 7:9083–91. 25924183 10.1039/c4nr05742b

[CIT0009] de la Fuente JM, Alcántara D, Eaton P, et al. (2006). Gold and gold-iron oxide magnetic glyconanoparticles: synthesis, characterization and magnetic properties. J Phys Chem B 110:13021–8. 16805609 10.1021/jp062522s

[CIT0010] de la Fuente JM, Barrientos AG, Rojas TC, et al. (2001). Gold glyconanoparticles as water-soluble polyvalent models to study carbohydrate interactions. Angew Chem Int Ed 40:2257–61. 10.1002/1521-3773(20010618)40:12<2257::AID-ANIE2257>3.0.CO;2-S 29711834

[CIT0011] El-Boubbou K, Gruden C, Huang X (2007). Magnetic glyco-nanoparticles: a unique tool for rapid pathogen detection, decontamination, and strain differentiation. J Am Chem Soc 129:13392–3. 17929928 10.1021/ja076086e

[CIT0012] Farr TD, Lai CH, Grünstein D, et al. (2014). Imaging early endothelial inflammation following stroke by core shell silica superparamagnetic glyconanoparticles that target selectin. Nano Lett 14:2130–4. 24564342 10.1021/nl500388h

[CIT0013] Fasman EGD, Gerald D. (1975). Handbook of biochemistry and molecular biology: lipids, carbohydrated, steroids. Florida, USA: CRC Press.

[CIT0014] Gallegos-Tabanico A, Sarabia-Sainz JA, Sarabia-Sainz HM, et al. (2017). Molecular recognition of glyconanoparticles by RCA and *E. coli* K88-designing transports for targeted therapy. Acta Biochim Pol 64:671–7. 29247504 10.18388/abp.2017_1639

[CIT0015] Gallo J, García I, Genicio N, et al. (2011). Specific labelling of cell populations in blood with targeted immuno-fluorescent/magnetic glyconanoparticles. Biomaterials 32:9818–25. 21940045 10.1016/j.biomaterials.2011.09.010

[CIT0016] García I, Gallo J, Genicio N, et al. (2011). Magnetic glyconanoparticles as a versatile platform for selective immunolabeling and imaging of cells. Bioconjug Chem 22:264–73. 21247095 10.1021/bc1003923

[CIT0017] García I, Marradi M, Penadés S (2010). Glyconanoparticles: multifunctional nanomaterials for biomedical applications. Nanomedicine (Lond) 5:777–92. 20662648 10.2217/nnm.10.48

[CIT0018] Grillon C, Matejuk A, Nadim M (2012). News on microenvironmental physioxia to revisit skin cell targeting approaches. Exp Dermatol 21:723–8. 22882247 10.1111/j.1600-0625.2012.01551.x

[CIT0019] Hakomori S (2001). Tumor-associated carbohydrate antigens defining tumor malignancy: basis for development of anti-cancer vaccines. Adv Exp Med Biol 491:369–402. 14533809 10.1007/978-1-4615-1267-7_24

[CIT0020] Han E, Ding L, Ju H (2011). Highly sensitive fluorescent analysis of dynamic glycan expression on living cells using glyconanoparticles and functionalized quantum dots. Anal Chem 83:7006–12. 21809847 10.1021/ac201488x

[CIT0021] Jafari MS, Khoshchehreh R, Goodarzi N, et al. (2014). *cis*-Dichlorodiamminoplatinum (II) glyconanoparticles by drug-induced ionic gelation technique targeted to prostate cancer: preparation, optimization and in vitro characterization. Colloids Surf B Biointerfaces 122:350–8. 25078298 10.1016/j.colsurfb.2014.06.065

[CIT0022] Jiang X, Ahmed M, Deng Z, et al. (2009). Biotinylated glyco-functionalized quantum dots: synthesis, characterization, and cytotoxicity studies. Bioconjug Chem 20:994–1001. 19402705 10.1021/bc800566f

[CIT0023] Kasteren SIV, Campbell SJ, Serres S, et al. (2009). Glyconanoparticles allow pre-symptomatic in vivo imaging of brain disease. Proc Natl Acad Sci U S A 106:18–23. 19106304 10.1073/pnas.0806787106 PMC2607245

[CIT0024] Kekkonen V, Lafreniere N, Ebara M, et al. (2009). Synthesis and characterization of biocompatible magnetic glyconanoparticles. J Magn Magn Mater 321:1393–6.

[CIT0025] Kikkeri RV, Bavireddi H (2012). Glyco-[small beta]-cyclodextrin capped quantum dots: synthesis, cytotoxicity and optical detection of carbohydrate–protein interactions. Analyst 137:5123–7. 23001235 10.1039/c2an35983a

[CIT0026] Kulkarni AA, Fuller C, Korman H, et al. (2010). Glycan encapsulated gold nanoparticles selectively inhibit shiga toxins 1 and 2. Bioconjug Chem 21:1486–93. 20669970 10.1021/bc100095w PMC3024884

[CIT0027] Liu S, Shi F, Zhao X, et al. (2013). 3-Aminophenyl boronic acid-functionalized CuInS2 quantum dots as a near-infrared fluorescence probe for the determination of dopamine. Biosens Bioelectron 47:379–84. 23608539 10.1016/j.bios.2013.03.055

[CIT0028] Marradi M, Martín-Lomas M, Penadés S (2010). Glyconanoparticles polyvalent tools to study carbohydrate-based interactions. Adv Carbohydr Chem Biochem 64:211–90. 20837200 10.1016/S0065-2318(10)64005-X

[CIT0029] Mirkin G (1973). Carbohydrate loading: a dangerous practice. JAMA 223:1511–12. 10.1001/jama.1973.03220130055023 4740039

[CIT0030] Nagahori N, Abe M, Nishimura SI (2009). Structural and functional glycosphingolipidomics by glycoblotting with aminooxy-functionalized gold nanoparticle. Biochemistry 48:583–94. 19117481 10.1021/bi801640n

[CIT0031] Nieman DC (1998). Influence of carbohydrate on the immune response to intensive, prolonged exercise. Exerc Immunol Rev 4:64–76. 9644095

[CIT0032] Ojeda R, de Paz JL, Barrientos AG, et al. (2007). Preparation of multifunctional glyconanoparticles as a platform for potential carbohydrate-based anticancer vaccines. Carbohydr Res 342:448–59. 17173881 10.1016/j.carres.2006.11.018

[CIT0033] Park S, Kim G-H, Park S-H, et al. (2015). Probing cell-surface carbohydrate binding proteins with dual-modal glycan-conjugated nanoparticles. J Am Chem Soc 137:5961–8. 25939670 10.1021/jacs.5b00592

[CIT0034] Qian R-C, Lv J, Li H-W, Long Y-T (2017). Sugar-coated nanobullet: growth inhibition of cancer cells induced by metformin loaded glyconanoparticles. ChemMedChem 12:1823–7. 28967197 10.1002/cmdc.201700583

[CIT0035] Schofield CL, And RAF, Russell DA (2007). Glyconanoparticles for the colorimetric detection of cholera toxin. Anal Chem 79:1356–61. 17297934 10.1021/ac061462j

[CIT0036] Sundgren A, Barchi JJ Jr. (2008). Varied presentation of the Thomsen-Friedenreich disaccharide tumor-associated carbohydrate antigen on gold nanoparticles. Carbohydr Res 343:1594–604. 18502409 10.1016/j.carres.2008.05.003 PMC2526251

[CIT0037] Sundhoro M, Park J, Jayawardana KW, et al. (2017). Poly(HEMA-*co*-HEMA-PFPA): synthesis and preparation of stable micelles encapsulating imaging nanoparticles. J Colloid Interface Sci 500:1–8. 28395159 10.1016/j.jcis.2017.03.099

[CIT0038] Töpfer-Petersen E (1999). Carbohydrate-based interactions on the route of a spermatozoon to fertilization. Hum Reprod Update 5:314–29. 10465523 10.1093/humupd/5.4.314

[CIT0039] Wang X, Matei E, Deng L, et al. (2013). Sensing lectin–glycan interactions using lectin super-microarrays and glycans labeled with dye-doped silica nanoparticles. Biosens Bioelectron 47:258–64. 23584388 10.1016/j.bios.2013.03.014 PMC3661755

[CIT0040] Wang X, Ramstro¨m O, Yan M (2010). Quantitative analysis of multivalent ligand presentation on gold glyconanoparticles and their effects on protein binding. Anal Chem 82:9082–9. 20942402 10.1021/ac102114z PMC3033484

[CIT0041] Won S, Hindmarsh S, Gibson MI (2018). Triggerable multivalent glyconanoparticles for probing carbohydrate-carbohydrate interactions. ACS Macro Lett 7:178–83. 29657901 10.1021/acsmacrolett.7b00891 PMC5894439

[CIT0042] Wu L, Zhang Y, Li Z, et al. (2017). “Sweet” architecture-dependent uptake of glycocalyx-mimicking nanoparticles based on biodegradable aliphatic polyesters by macrophages. J Am Chem Soc 139:14684–92. 28950065 10.1021/jacs.7b07768

[CIT0043] Yang Y, Xue XC, Jin XF, et al. (2012). Synthesis of multivalent *N*-acetyl lactosamine modified quantum dots for the study of carbohydrate and galectin-3 interactions. Tetrahedron 68:7148–54.

[CIT0044] Yang Y, Yu M, Yan TT, et al. (2010). Characterization of multivalent lactose quantum dots and its application in carbohydrate-protein interactions study and cell imaging. Bioorg Med Chem 18:5234–40. 20566293 10.1016/j.bmc.2010.05.046

[CIT0045] Yang Y, Zhao YT, Yan TT, et al. (2010). Design and fabrication of multivalent gal-containing quantum dots and study of its interactions with asialoglycoprotein receptor (ASGP-R). Tetrahedron Lett 51:4182–5.

[CIT0046] Zhou J, Hao N, Zoyza TD, et al. (2015). Lectin-gated, mesoporous, photofunctionalized glyconanoparticles for glutathione-responsive drug delivery. Chem Commun 51:9833–6. 10.1039/c5cc02907d PMC4456355 25989158

